# Agricultural diversification in West Africa: an archaeobotanical study of the site of Sadia (Dogon Country, Mali)

**DOI:** 10.1007/s12520-021-01293-5

**Published:** 2021-03-08

**Authors:** Louis Champion, Dorian Q. Fuller, Sylvain Ozainne, Éric Huysecom, Anne Mayor

**Affiliations:** 1 Laboratoire Archéologie et Peuplement de l’Afrique (APA), Anthropology Unit of the Department of Genetics and Evolution, University of Geneva, 30 quai Ernest Ansermet, CH-1205 Geneva, Switzerland; 2 Royal Museums for Central Africa, Tervuren, Belgium; 3 Institute of Archaeology, University College, London, UK; 4 School of Cultural Heritage, Northwest University, Xi’an, Shaanxi China; 5 Department of Archaeology, Max Planck Institute for the Science of Human History, Jena, Germany; 6 Office cantonal d’Archéologie, Canton du Valais, Switzerland; 7 Global Studies Institute, University of Geneva, Geneva, Switzerland

**Keywords:** Archaeobotany, Agriculture, Food diversification, Fonio, Rice, West Africa

## Abstract

While narratives of the spread of agriculture are central to interpretation of African history, hard evidence of past crops and cultivation practices are still few. This research aims at filling this gap and better understanding the evolution of agriculture and foodways in West Africa. It reports evidence from systematic flotation samples taken at the settlement mounds of Sadia (Mali), dating from 4 phases (phase 0=before first–third century AD; phase 1=mid eighth–tenth c. AD; phase 2=tenth–eleventh c. AD; phase 3=twelfth–late thirteenth c. AD). Flotation of 2200 l of soil provided plant macro-remains from 146 archaeological samples. As on most West African sites, the most dominant plant is pearl millet (*Pennisetum glaucum*). But from the tenth century AD, sorghum (*Sorghum bicolor*) and African rice (*Oryza glaberrima*) appear in small quantities, and fonio (*Digitaria exilis*) and barnyard millet/hungry rice (*Echinochloa* sp.), sometimes considered weeds rather than staple crops, are found in large quantities. Some samples also show remains of tree fruits from savannah parklands, such as baobab (*Adansonia digitata*), marula (*Sclerocarya birrea*), jujube (*Ziziphus* sp.), shea butter (*Vittelaria paradoxa*) and African grapes (*Lannea microcarpa*). Fonio and *Echinochloa* sp. cultivation appears here to be a later addition that helped to diversify agriculture and buffer against failures that might affect the monoculture of pearl millet. This diversification at the end of the 1st millennium AD matches with other evidence found in West Africa.

## Introduction

Food and drink form a foremost concern of all societies. Much of human energy is spent fuelling nutritional needs and tastes. Productivity of agriculture may ultimately determine population density, community size, and rate at which growing populations expand through migration (e.g. Fuller et al. 2019). Beyond subsistence needs, food is also crucial for structuring social relationships, reinforcing cultural identity and marking the calendar through seasonal feasts (Hastorf 2016: 142ff; Twiss 2019: 98ff). Feasting event and beer production are usually required by social conventions, and from African ethnographic examples it is estimated that 15–20% of agricultural production goes towards such productions (Dietler 2001: 81–82). Archaeobotanical evidence provide empirical data on past productive systems, from which estimates of the productive capacity and the potential raw materials for daily cuisine and feasts can be reconstructed (Mercuri et al. 2018a; Champion and Fuller 2019).

**Fig. 1:**
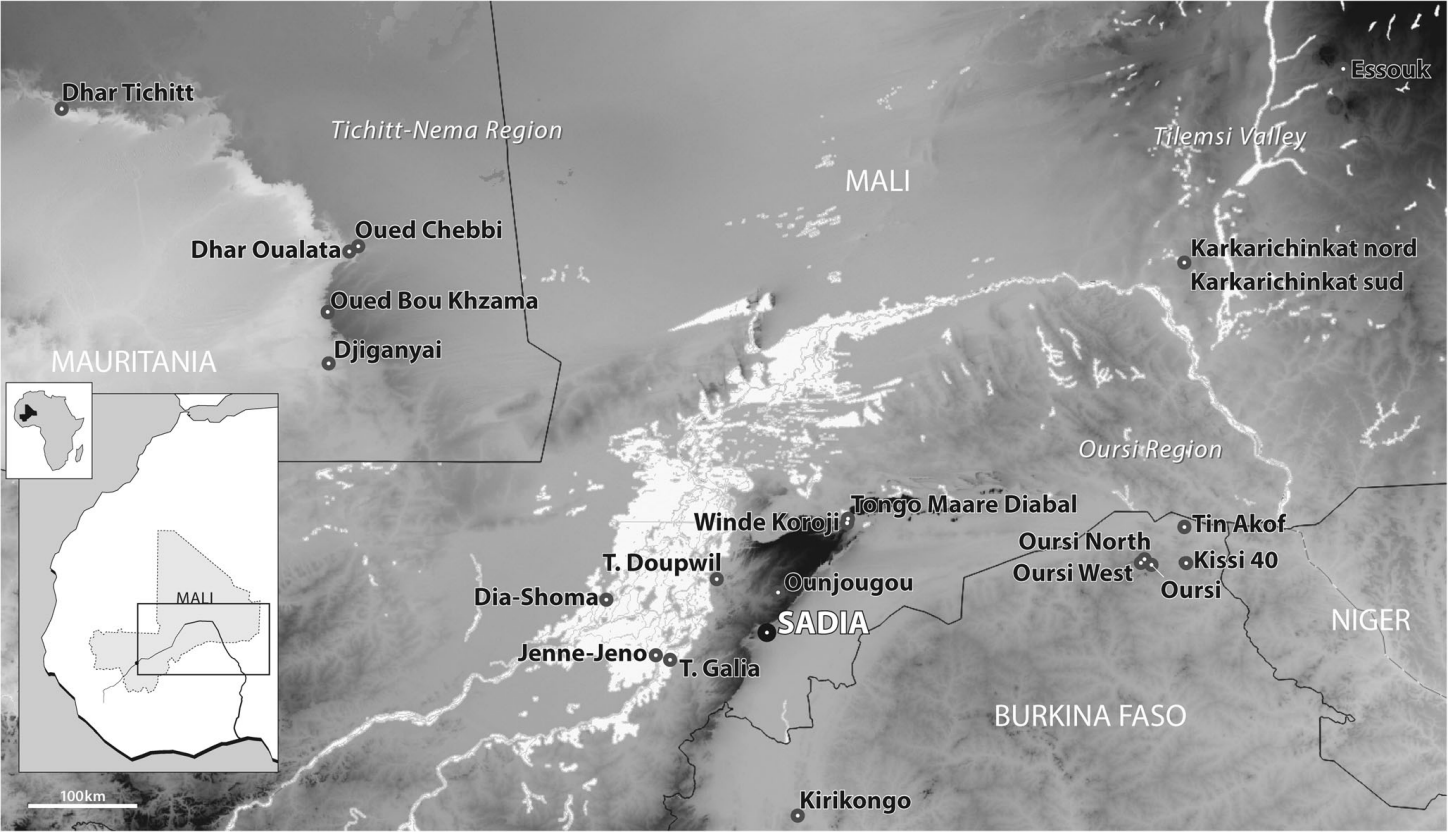
Map of the sites cited in the text (based on the database of Champion 2019)

The aim of this paper is to contribute new empirical evidence that documents some of the evolution of West African food traditions during the past two millennia through the analysis of an important assemblage of archaeobotanical samples coming from the excavations of the stratified and well-dated settlement site of Sadia in Mali (Fig. 1). A key issue is the timing and extent of agricultural diversification in Africa, as crops such as African rice (*Oryza glaberrima* Steud.), fonio (*Digitaria exilis* (Kippist) Stapf.), cowpea (*Vigna unguiculata*) and sorghum (*Sorghum bicolor* (L.) Moench) helped to expand the agricultural repertoire beyond the Neolithic reliance on pearl millet (*Pennisetum glaucum*) (Kahlheber and Neumann 2007; Champion and Fuller 2018a; Kay et al. 2019).

## Archaeological background

### Localisation and current environment

The archaeological site of Sadia, located in the Seno Plain in Central Mali, is composed of five anthropogenic mounds covering an approximate area of 3 ha on a small natural elevation. It is settled along the Guringin Valley, perpendicular to the Bandiagara escarpment.

Archaeological work was part of an extensive interdisciplinary study of the Dogon Country conducted within the project ‘Human population and Palaeoenvironment in Africa’ under the leadership of E. Huysecom (Huysecom et al. 2004; Mayor 2011 b; Mayor et al. 2005, 2014; Ozainne 2013; Ozainne et al. 2017). Extensive surveys and small-scale test pits were first conducted along the Guringin valley (Loukou et al. 2013), and then two campaigns of excavations took place at the settlement site of Sadia in 2010 and 2011, the first one focusing on a vertical exploration of four mounds, and the second one on a spatial exposure at the summit of the main mound (Huysecom et al. 2011, 2012, 2015).

In the region, modern rainfall reaches 500–600 mm per annum, but probably varied in the past and was higher during the time of Sadia’s occupation (Mayor et al. 2005; Garnier et al. 2015). The environment is today a largely open Sahelian grassland with scattered trees and shrubs. A long history of pastoralism has encouraged high proportions of spiny taxa like *Acacia*, while selective cutting, tree management and possibly planting has helped to create a parkland of many useful tree species like *Faidherbia albida*, *Vitellaria paradoxa*, *Adansonia digitata*, *Borassus aethiopum*, *Cordyla pinnata*, *Tamarindus indica* and *Sclerocarya birrea* (Boffa 1999; Höhn and Neumann 2012; Taïbi 2019). Several of these trees produce edible fruits.

Traditional local agriculture focuses on pearl millet, sorghum and minor millets such as fonio as carbohydrate staples, with various vegetables and pulses, such as cowpea (*Vigna unguiculata* (L.) Walp.), as companion crops. Occasional wild rice (e.g. *Oryza barthii*) and various other wild grasses gathering has been documented in the region, namely during periods of starvation or bad success of agriculture (Selleger 2014; personal inquiries). Currently, rice cultivation is mostly concentrated to the wetter setting in the Niger valley, such as the Inland Delta, and in the Sourou valley at the border with Burkina Faso. However, rice is also cultivated in the Dogon country, but represents only a very small contribution to local agriculture.

### Chrono-stratigraphy

**Fig. 2:**
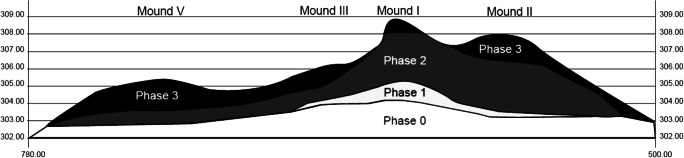
Synthesised transects showing the extension of each phase in the four investigated mounds of Sadia (vertical scale magnified 7.5 times; From Huysecom et al. 2015:12)

A total of 27 radiocarbon dates analysed with Bayesian modelling has enabled us to build a chrono-cultural framework of the site of Sadia, extending from about 750 to 1300 AD (Huysecom et al. 2011, 2012, 2015). The mounds’ development is preceded by a pre-tell occupation (phase 0) in a yellow sandy layer dated from a single radiocarbon date (1895±35 BP, ETH-40328) as a terminus ante quem of first to third century AD. The tell occupation itself includes three main phases (Fig. 2):

- Phase 1 witnesses the foundation of the village of Sadia at the location of the most important mound of the site (mound I). It is dated from the middle of the eighth to the tenth century AD.
- Phase 2 represents the most important growth of the site to as large as 1.5 ha which occurred mainly during the tenth and eleventh c. AD.
- Phase 3 reaches a maximum area of approximately 3 ha during the twelfth and thirteenth c. AD, before the site is abandoned. Archaeological dwelling structures are well preserved, despite some disturbance of trees. As the summit of the site had been exposed to erosion, the exact date of its abandonment is unknown, but it is suggested that this occurred around 1300 AD.

The stratigraphy indicates a continuous occupation, without any interruptions or destructive layers.

### Important features

**Fig. 3:**
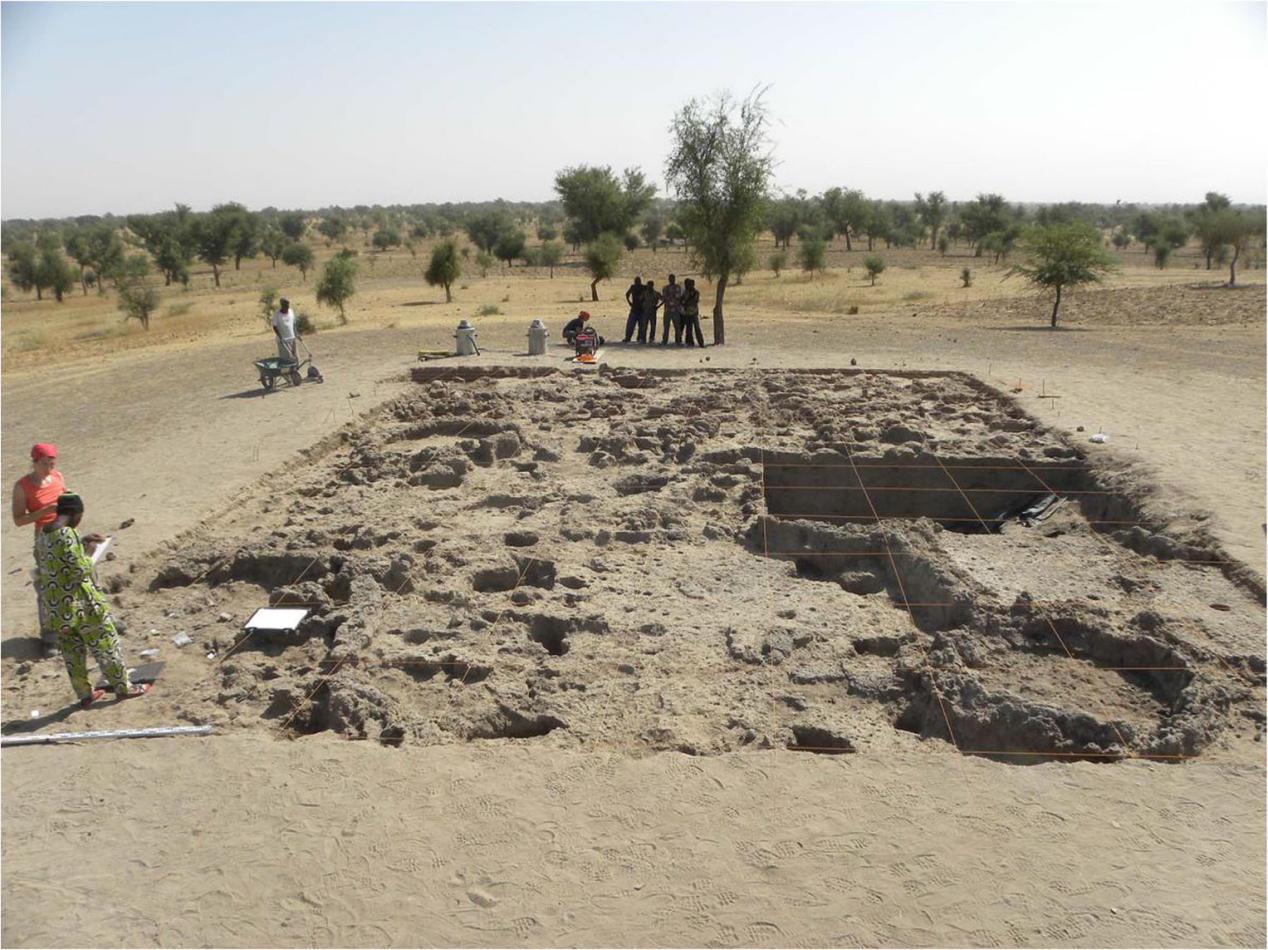
View of Sadia mound I extensive excavation showing architectural features of phase 3 (photo APA 2011)

**Fig. 4:**
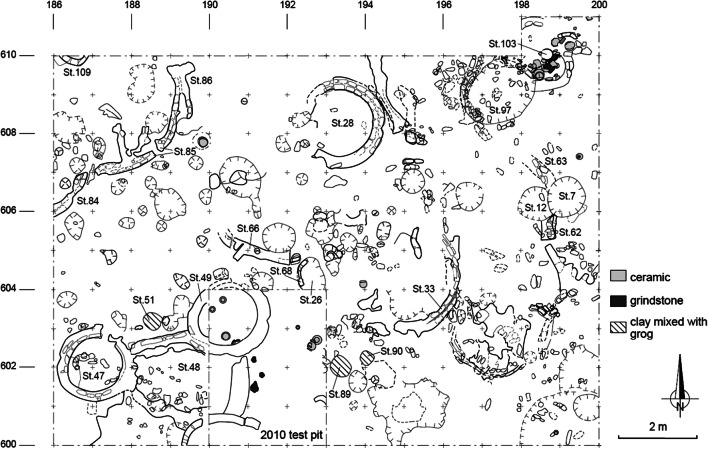
Plan of Sadia mound I extensive excavation. Overview of architectural features of phase 3 (from Huysecom et al. 2015:15)

Extensive excavations of 143 m^2^ conducted on the top of mound I allowed a better knowledge of the architectural characteristics of phase 3 (Figs. 3 and 4). Around 200 structures were found and revealed domestic units composed of circular rooms and spaces of different shapes limited by strait walls. Some of the circular rooms are too small for houses and were probably related to specific activities such as storing or cooking. Most of the buildings were made with mud bricks. Occupation floors, post holes, refuse pits, hearths and two burials of children were also found in deeper layers (Huysecom et al. 2015).

Animal remains studied by Louis Chaix (Museum of Natural History in Geneva) are mainly based on domesticates. Caprine remains are the largest part of the livestock (51%) with goat (*Capra*) being more important than sheep (*Ovis*). Cattle (*Bos Taurus*) represent around 30% of the animal remains. Donkeys (*Equus asinus*) and chickens (*Gallus gallus*) are present in small quantities. Wild animals (<5%) are represented by varieties of gazelles, giant rats, birds and reptiles (crocodiles, frogs and snakes). Fish are well-represented by species found in floodplains and temporary ponds. Diachronic analysis has shown a reduction in domesticates with a decrease of cattle that parallels an increase of caprines through the sequence (Huysecom et al. 2015).

Besides objects of local production like ceramics, iron objects or grindstones, some goods from medium- and long-distance trade were also found, such as copper jewellery and some carnelian and glass beads (Huysecom et al. 2015). Chemical analysis of one glass bead (eleventh–thirteenth c. AD) indicated a composition like the ones of beads discovered in the Iberian Peninsula and Southern Africa, but with an origin of the glass still unknown. These results highlight the trade connections between the Seno plain, the Niger Bend and the major trans-Saharan trade routes (Truffa Giachet 2019; Truffa Giachet et al. 2020).

Regional surveys have shown that many settlements of similar size were established in the Seno plain, suggesting the development of non-centralised agro-pastoral societies during a peaceful period. The turn of the thirteenth to the fourteenth century AD witnessed a large abandonment of the sites in the region, including Sadia, but also in most of West Africa. From abrupt climate changes to war, invasion (i.e. Songhay) and diseases (i.e. the plague), several reasons are proposed for this sub-continental settlement rupture. However, no strong evidence can confirm or refute any of the reasons yet (Huysecom et al. 2015; Champion 2019).

## Material and methods

During excavations, Stephanie Kahlheber (University of Frankfurt) and colleagues from the Mission Culturelle de Bandiagara collected systematically archaeobotanical samples within the deep test pits in 2010 and in the horizontal excavation on mound I in 2011, where almost each context was sampled, except the wall remains. The average amount of soil collected was 15 l for each sample, but some smaller contexts, such as hearths, were collected in their entirety.

In total, around 2200 l of archaeological soil matrix coming from 146 samples was processed by bucket flotation directly in the field. Flotation is a method widely used to separate materials of different density, and is the most common way of recovering charred plant remains from archaeological contexts, as soil particles sink and charred plant remains float (Nesbitt 1995). Whether mechanical (using a flotation drum or machine) or manual (using buckets), the basic principle is to mix the sediment with water to wet it and break it apart. The buoyant material—including the plant macro-remains—is then poured off and collected in a fine mesh (Fuller 2008; Champion and Fuller 2019). The weight and volume of each flot was then measured, and each sample was sieved into five size fractions: >4 mm, 2–4 mm, 1–2 mm, 0.5–1 mm, <0.5 mm. The ≥0.5 mm sub-samples were sorted for charred plant elements such as seeds, fruits, and parenchyma with the aid of a low-power stereomicroscope (×10–40).

Identification of cereals, chaff, pulses, millets, weeds, nuts and fruits was made by comparing archaeological morphotypes with taxonomic specimens in the modern reference collection housed at the Institute of Archaeology, University College London, and isolate samples at the University of Geneva. Taxa identification follows the nomenclature of The Useful Plants of West Tropical Africa (Burkill 1985–2000). Photographs and descriptions of identification from a variety of published literature were also used (Fuller 2003; Fuller and Harvey 2006; Nixon 2011; Murray 2000, 2007; D’Andrea et al. 2001). For all the samples analysed, whole vegetal items were recorded by count in a list of taxa, from which tables of relative frequency were constructed, and ubiquity calculated. The relative frequency is the percentage of total seed count. It can be calculated by sample, phase, trench or site, as necessary. In addition, every taxon was logged in a ubiquity table. The ubiquity is the number of samples in which a taxon is present as a percentage of the total number of samples.

## Results

**Fig. 5:**
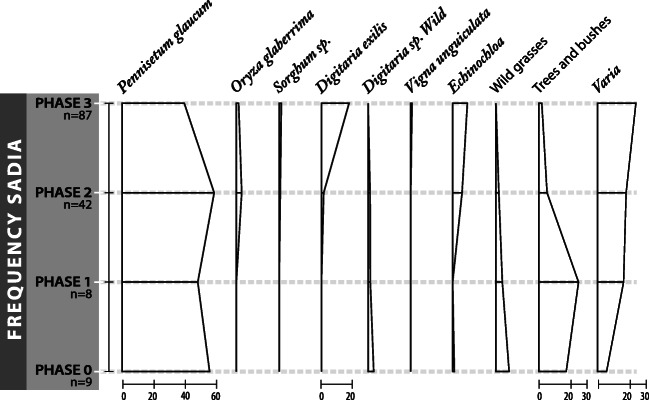
Frequency (% of remains of a species compared with the whole period assemblage) for the archaeobotanical dataset at Sadia, including 146 samples (*n* = number of samples by phase)

**Fig. 6:**
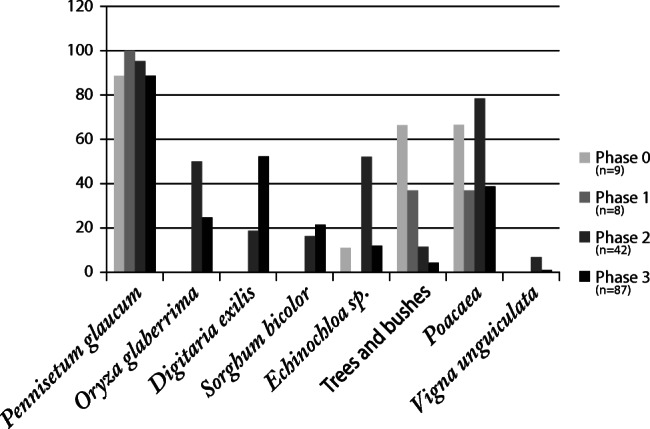
Ubiquity (% of samples by phase in which each taxon is present) for major crops at Sadia

Here we present the results of our analysis of seeds from all samples, integrating a previous preliminary analysis by B. Eichhorn and S. Kahlheber including grains and wood charcoal, which was published elsewhere (Huysecom et al. 2012, 2015). Of the 146 collected samples analysed here, only five were entirely devoid of identifiable macro-botanical remains. In total, around 10,104 plant remains were recorded within 141 samples for an average density of 4.5 remains per litre of soil. Almost all the remains comprise carbonised seeds, although some silicified African rice husks (mainly lemma) were found, and these are also included in the counts in Table 1, and Figs. 5 and 6, that provide frequency and ubiquity of important species.

**Table 1:** Number of items recovered, frequency and ubiquity for the main crops from Sadia by phases. First line is the total number of items and second line gives frequency followed by ubiquity

Identified species	Phase 0 ‘pre-tell’	Phase 1 8–10th c.	Phase 2 10–11th c.	Phase 3 12–13th c.	Total
Number of samples Volume of soil in litres	9 111	8 86	42 666	87 1317	146 2200
*Pennisetum glaucum* caryopsis	73	36	1015	695	1819
*Pennisetum glaucum* involucre	89	202	1428	1369	3088
*Pennisetum glaucum* total	162	238	2443	2064	4907
Frequency Ubiquity	56% 89%	49% 100%	59% 95%	40% 88%	48% 91%
*Digitaria exilis* caryopsis	–	–	37	931	968
Frequency Ubiquity	–	–	1% 19%	18% 53%	10% 40%
*Echinochloa* sp. caryopsis	2	–	237	480	719
Frequency Ubiquity	1% 11%	–	6% 52%	9% 13%	7% 23%
*Oryza glaberrima* caryopsis	–	–	6	6	12
*Oryza glaberrima* Spikelets bases	–	–	108	59	167
*Oryza glaberrima* total	–	–	114	65	179
Frequency Ubiquity	–	–	3% 50%	1.5% 25%	2% 29%
*Vigna unguiculata* seed	–	–	9	1	10
Frequency Ubiquity	–	–	<1% 7%	<1% 1%	1% 3%
*Sorghum bicolor* caryopsis	–	–	10	47	57
*Sorghum bicolor* spikelet base	–	–	10	23	33
*Sorghum bicolor* total	–	–	20	70	90
Frequency Ubiquity	–	–	<1% 17%	<1% 22%	<1% 18%
*Hibiscus cf. sababdariffa* caryopsis	5	–	29	–	34
Frequency Ubiquity	2% 22%	–	<1% 11%	–	<1% 5%
*Poaceae* sp. grain	20	20	49	37	126
Frequency Ubiquity	7% 67%	4% 38%	2% 12%	<1% 5%	1% 12%
Weeds and/or wild gathered plants	20	13	142	124	299
Frequency Ubiquity	7% 78%	3% 50%	4% 43%	3% 29%	3% 37%
Tree/bush	50	125	214	90	479
Frequency Ubiquity	17% 67%	26% 38%	5% 79%	2% 39%	5% 52%
Total number	288	489	4114	5213	10104
Density items/litre	2.6	5.7	6.2	3.9	4.6

The archaeobotanical results indicate that the principal plant remains from Sadia are domesticated pearl millet, followed by fonio and *Echinochloa* sp. remains. Other domesticated crops are also present but in lower frequency: African rice, sorghum, *Hibiscus* and cowpea. The consumption of fruits of local trees and wild gathered grasses is also indicated.

This section presents a description of each of the main economic taxa found at Sadia, an analysis of their evolution through the four phases of the site occupation, and a spatial analysis for phase 3 on mound I.

### Description of the main economic taxa at Sadia

#### Pearl millet, *Pennisetum glaucum* (L.) R. Br., Poaceae

**Fig. 7:**
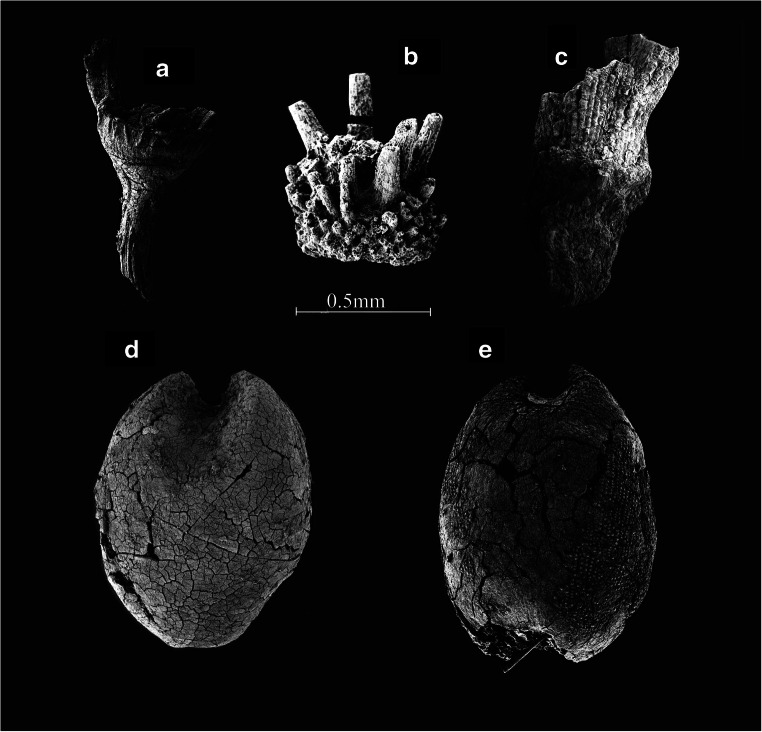
SEM micrographs of Plant remains from Sadia. (**a**) *Sorghum bicolor* spikelet base with torn rachilla, AB 38 St 60. (**b**) *Pennisetum glaucum* involucres apex with bristles, AB 11 St26. (**c**) *Oryza glaberrima* spikelet base, AB 11 St26. (**d**) *Digitaria exilis* grain Embryo face, AB 4 St4. (**e**) *Digitaria exilis* grain Hilum face, AB 4 St4

Pearl millet is represented by 4907 remains (chaff and grains). Pearl millet is present in 91% of the samples and constitutes 48% of the total frequency. The seeds represent 37% (1819 grains) of the millet assemblage, while chaff (bristles and involucre bases, B in Fig. 7) represent 63% (3088) (Table 1). Due to their morphological characteristics, club-shaped grains and stalked involucres, these are all determined as domesticated pearl millet (after Brunken et al. 1977; Manning et al. 2011).

#### Fonio, *Digitaria exilis* (Kippist.) Staph., Poaceae.

A total of 968 fonio grains, 10% of the total assemblage, were found in 40% of the samples (Table 1, D and E in Fig. 7). Fonio is a small-seeded millet, around 1.2 × 0.7 × 0.5 mm. The grain form is ovoid to oblong with an ovoid scutellum, less than half the length of the grain, and a roundish hilum on the ventral surface (Stapf 1915; Portères 1976).

#### Barnyard millet/hungry rice, *Echinochloa* sp., Poaceae

*Echinochloa* sp. is the third most important plant at Sadia. It is well represented with 719 caryopses representing 7% frequency and 23% ubiquity. In West Africa, *Echinochloa* sp. is represented by five different species. The grains present very similar characteristics making it difficult to distinguish one species from another.

#### African Rice, *Oryza glaberrima* Steud., Poaceae

African rice was found in 43 samples and represented 2% of the total assemblage (Table 1). This includes 12 grains and 167 spikelet bases (C in Fig. 7) and hundreds of husks (lemma/palea fragments).

Rice spikelet bases provide a good way to differentiate wild and domesticated rice. One of the most important key effects of plant domestication is a shift from natural seed dispersal to human seed dispersal. Mature wild grains fall naturally from the plant creating a smooth, round and regular abscission scar with a small distinct vascular port on the spikelet base. On the opposite, domesticated non-shattered grain needs human threshing to fall apart. The result of this labour is to create a less symmetrical scar with a dimpled appearance (Fuller et al. 2009; Fuller and Weisskopf 2011; Ishii et al. 2010). It is observed that within the 167 spikelet bases of the Sadia dataset, 60% are domesticated and only 6.5% are wild. A further 12% are classified as immature and, due to their bad preservation, the remaining 22% could not be determined. Excluding immature and indeterminate forms, the domesticated proportion can thus be estimated as ~90%. These data indicate that rice at Sadia was from already domesticated populations, with a small presence (~10%) of wild-type or weedy rice (*O. barthii*).

#### Sorghum, *Sorghum bicolor* subsp. *bicolor* (L.) Moench., Poaceae.

Only 90 (less than 1% of the total assemblage) sorghum remains, mainly grains (57) but also chaff (33), were found in 18% of the samples (Table 1). The chaff is composed of husks (lemma/palea), spikelet bases (A in Fig. 7), with the torn rachilla characteristic of domesticated sorghum. Such remains probably come from the dehusking waste of race bicolor (on races, see Fuller and Stevens 2018).

#### Cowpea, *Vigna unguiculata* (L.) Walp., Fabaceae

In Sadia, cowpea remains are few, only 10 seeds (representing less than 1% of the total assemblage) were found. The seeds are flat with a sub-rectangular shape. The hilum is ovate and generally placed asymmetrically on the hilum edge (Fuller 2003; Fuller and Harvey 2006).

#### Roselle, *Hibiscus sabdariffa* Lin., Malvaceae

In total, 34 Roselle caryopses, representing less than 1% of the assemblage, were found. The identification was done following the criteria published by Kahlheber (2004).

#### Fruits, tree and bushes

In total, 479 remains of fruits, 5% of the total assemblage, were found in 52% of the samples (Table 1). Charred remains include baobab (*Adansonia digitata* L.), marula (*Sclerocarya birrea* A. Rich. syn. *Poupartia birrea* (A. Rich.) A. Chev.), jujube (*Ziziphus* sp.), shea butter (*Vittelaria paradoxa* Gaertn. f.), African grapes (*Lannea acida* A. Rich. Syn. *L. microcarpa* Engl. & Krause) and tamarind (*Tamarindus indicus* L.).

#### Weeds and/or wild gathered plants

A number of seeds of known weed taxa (299 seeds for 3% of the total assemblage in 37% of the samples) were recovered (Table 1). They include carpet weeds (*Trianthema* sp., family Aizoaceae) and button weeds (*Borreira* sp./*Spermococce* sp., family Rubiaceae), characterised by intermediate size, large-seeded weedy grasses (*Paspalum* and *Echinochloa*), and other monocots (*Commelina* spp.), characterised by quite large and heavy seeds.

#### Indeterminate Poaceae species

One hundred twenty-six Poaceae grains, undetermined to genus/species (1% frequency), were found in 12% of the samples. Because of the lack of reference collections complete for African grasses and due to preservation and damaged specimens (most of the caryopses were without hilum), it was not possible to make a more precise identification.

### Diachronic analysis

The 146 samples studied have provided plant remains from four archaeological phases at Sadia. The diachronic distribution of archaeobotanical remains is summarised in Table 1, and Figs. 5 and 6.

#### Phase 0: ‘pre-tell phase’—before first–third century AD

Phase 0 is an occupation episode older than the occupation of the mound site. It is represented by nine archaeobotanical samples, totalling 111 l of sediment from mound I. As expected, pearl millet (*Pennisetum glaucum*) dominates identified remains, with a frequency of 56% for 162 remains of grain and involucres. *Hibiscus sabdariffa* is the only other cultivated plant of this phase, present with 5 seeds (≈ 2% of the assemblage).

Interestingly, there are no evidence of other cereals, either sorghum or fonio, but remains of weeds and gathered seeds (wild seeds) are well represented by around 7%. This category is dominated by *Digitaria* sp*.* seeds (elongated form, wild-type), which could be a wild gathered precursor to fonio, and by button weeds, *Borreria* sp*.* One single wild African rice spikelet base, *Oryza barthii* type, was also found. In addition, 9% of wild grasses (Poaceae) are present. With 17%, trees and shrubs are also well represented with various taxa: Marula tree (4%), baobab (4%), tamarind (≈ 1%), jujube (≈2%), shea butter nut tree (≈ 3%) and black plum (1%).

#### Phase 1: mid eighth–tenth c. AD

For the foundation phase of the tell of Sadia, only eight archaeobotanical samples, of 86 litres of sediment, have been acquired. The assemblage is composed of around 50% of pearl millet. A decrease of ‘weeds and gathered’ is visible: from 7% in phase 0 (with a ubiquity of 78%), these drop to less than 3%, with a presence in less than half of the samples in this phase. Only elongated wild *Digitaria* sp. and carpetweeds (*Trianthema* sp.) seeds are present. The trees and bushes (26%) are largely dominated by baobab remains with 17% of the total frequency and 87 seeds. This high frequency is explained by the presence of 69 large baobab seeds in only one of the samples.

#### Phase 2: tenth–eleventh c. AD

This period coincides with the most important expansion of the tell site. In this phase, the archaeobotanical results come from 42 samples: 23 from mound I, 12 samples from mound II and seven from mound III, comprising a total of 662 l of soil.

Once more, pearl millet dominates the dataset with 60% and 2443 remains (1015 grains and 1428 involucres). However, several other crops occur, including African rice (≈3%); sorghum (<1%); broad-seeded *Digitaria* (*Digitaria exilis*), identified as domesticated fonio (≈1%); and the pulse cowpea (<1%). In addition, seeds of *Echinochloa s*p*.* occur in a high quantity, around 6% of the total phase 2 frequency with 237 seeds. It is also noticeable that the weeds that appear for the first time in the assemblage in this period are all plausible rice weeds, such as *Commelina* cf. *benghalensis* (≈1%), *Oryza barthii* (<1%), *Trianthema* sp*.* (<1%), *Borreria* sp*.* (<1%), *Eleusine indica* (<1%), *Digitaria* sp. (1%) and *Paspalum* sp. (<1%). Beside this, presence of trees and shrubs dramatically decreased to ~5%, with a high proportion of marula nuts (around 3%).

#### Phase 3: twelfth–late thirteenth c. AD

Phase 3 is characterised by the end of the expansion and the abandonment of the site. It is well represented with a total of 87 samples from 1317 l of soil. This final phase has been sampled in two different ways: 22 samples were extracted during the 2010 vertical excavations in mound I (*n*=2), II (*n*=14) and III (*n*=6), and 65 samples come from the 2011 horizontal excavation of mound I.

With nearly one thousand grains representing 18% of all plant remains, spread across 53% of the samples, fonio represents an important crop during this period and a major increase over that seen in previous periods. Pearl millet remains still form the dominant taxon, but fall from 60% frequency, as seen in phase 2 (2443 remains in 95% of the samples), to less than 40% frequency (2064 remains in 88% of the samples) in phase 3. A reduction is seen across most of the assemblage in favour of fonio. The presence of *Echinochloa* also increases to more than 9% of the total in phase 3. Sorghum is rare (<1% of the phase frequency). African rice declines from 3 to 1%. Trees and bushes also decrease to less than 2%, with the presence of very few fruits of Marula, baobab or jujube (*Ziziphus* sp.) (less than 100 fruit fragments in total).

### Spatial distribution for phase 3

**Fig. 8:**
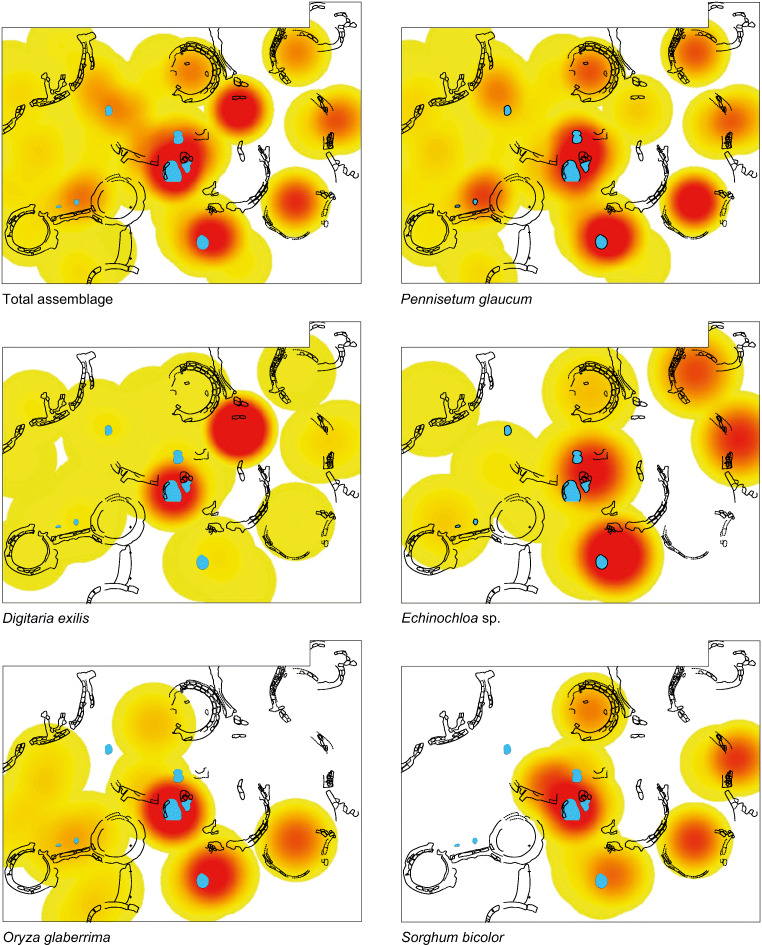
Spatial distribution of the density of the main cash crops at Sadia, mound I. The small circles represent the hearths

Spatial analysis may offer interesting insights into intra-site patterns that could relate to activity areas or differences in food use across the site (Fig. 8). This kind of analysis, however, is made difficult by the fact that preserved plant remains require charring for preservation. Thus, food processing, crop-processing and other activities, if waste is simply left in situ, will not be readily visible due to decomposition, unless the building has burned down. Instead, it must be recognised that the waste of food plant use activities must have been gathered up and disposed in fires after which some of this material survived charring and got redeposited nearby (Fuller et al. 2014). Nevertheless, broad patterns contrasting across building contexts or areas of a site may still be possible (e.g. Hastorf 1991).

The extensive horizontal surface of 143m^2^ excavated on Sadia mound I revealed numerous architectural features belonging to phase 3 (twelfth–thirteenth c. AD). Almost every structure, excluding wall remains, was sampled and around 3000 plant remains were extracted from 65 archaeobotanical samples, totalling 901 l of soil.

For the spatial analysis, densities were computed using QGIS 2.18.17 and the SAGA (2.3.2) Kernel density estimation tool, as well as quartic kernel and a 0.01-m cell resolution. The radius parameter was determined for each point dataset (i.e. each species and the total botanical samples assemblage) with an optimal bandwidth calculation based on the number of points and the spatial standard deviation, using the following formula: ((2/(3×nPoints))^(1/4))×standard deviation. Bandwidths vary between 1.37 m (total assemblage) and 1.88 m (*Echinochloa*).

The total assemblage distribution shows that remains are mainly found around the centre of the excavated area, while they appear in low amounts in the western part of the surface and show a more patchy pattern in the eastern part of the site. The separate analysis of the five hearth samples (small circles in Fig. 8) indicates similar proportions of the main crop remains as the other samples, with two exceptions. The first exception is *Echinochloa* sp. remains, which are not present inside the hearths but are often found in nearby structures. *Echinochloa* is also found in higher amounts in structure 7, which was interpreted as a silo pit (Huysecom et al. 2012). The second exception is fonio, which is mainly present in two specific places. One is structure 4, a pit that exclusively contained charred fonio grains that is 380 grains of the 968 grains from the total assemblage, representing 40% of the total fonio remains. The other is structure 26, a pit filled with a succession of dense ashy layers and carbonised wood and grain layers (see Fig. 4 for the numbered structures). This pit may contain refuse of the two hearths located close by. This group of structures including two hearths, one refuse pit and burnt clay bricks, full of domestic remains like animal bones and diversified charred plant remains may be interpreted as a kitchen area.

*Oryza* and *sorghum* also show particular spatial patterns, as *Oryza* mainly occurs in central and western part of the site, while *sorghum* is rather associated with the eastern part of the surface, in particular in structure 7. However, these specific distributions may be explained by sample/conservation biases, as both species are present in low amounts.

We assume here that remains were not massively translocated, but disposed into fires close to where crop-related activities took place, and then deposited after hearths were cleaned into nearby rubbish dumps. Consequently, precise activity areas are blurred, but broad patterns show that the different crops were not treated the same way in all areas of the settlement.

## Discussion

### Agricultural practices and food preparation

Based on modern ethnographic and agronomic observations as well as recent parallels (e.g. Portères 1976; Burnham 1980; Fuller et al. 2019; Champion 2019), the archaeobotanical remains indicate that Sadia’s inhabitants were practising primarily dry agriculture, possibly coupled with some wet rice agriculture in the later phase of the occupation. Sadia’s dry agriculture was probably composed of pearl millet and sorghum fields intercropped with cowpea. The field’s edges were probably, as it is often the case today, demarcated by sorrel (*Hibiscus*) cultivation. Fonio was likely grown in third or fourth years before fallowing or in areas of shallower soils. Fields were probably located in sandier soils at some distance from the Guringin river or from the eventual ponds, and the only water source was rainfall. The second type of agriculture found at Sadia was possibly related to the river itself. Indeed, the finds of African rice and *Echinochloa* sp. remains suggest that wet agriculture was also in practised around Sadia. Today, West African wet agricultural systems are dominated by rice with some décrue sorghum cultivation. Also, one of the most common weeds, especially in the Inland Niger Delta but also in the valley of the Sourou, only 60 km away, found growing in association with rice, is Bourgou grass, *Echinochloa stagnina*, which is gathered with the rice crop*.* Bourgou grass is also collected as fodder for livestock. However, *Echinochloa* sp. could be considered a cultivated crop as it may be an intended product from prepared fields, even if it lacks full domestication syndrome. It is fairly common that modern agricultural researchers consider small millet grasses as weeds rather than cultivars. This means that they have been under-researched in terms of intentional cultivation or domestication adaptations. It is worth noting that *Echinochloa* sp. has been domesticated several times in Asia (De Wet et al. 1983), and widely documented as gathered food grains (Harlan 1989; Yang et al. 2015). It has recently been shown to undergo selection and possible domestication also in the Middle Holocene Sahara (Mercuri et al. 2018b).

#### Pearl millet and sorghum

For sorghum and pearl millet, crop-husbandry processes are the same. The regular presence of pearl millet and sorghum remains in Sadia domestic contexts and the high involucre base/spikelet base numbers suggests that pearl millet and sorghum were stored as full panicles and were daily threshed when needed. Indeed, the bristle and involucre bases are usually separated from the grain during the threshing and winnowing steps of the crop processing. The high quantity of these elements found in each context and sample suggests that threshing and winnowing were processed in close relation with fires used for rubbish disposal, probably located not far from the household compounds. We infer that such crop-processing was a routine, household activity.

Pearl millet is one of the most common staple cereals used in Africa. Today, the seeds are generally transformed into porridge or beer (Jolly 2004; Haaland 2007; Ricquier 2014). So far, the first archaeological evidence of pearl millet consists of a couple of charred grains and seed impressions in ceramics that are dated to from ca. 2500 BC to 2000 BC and were found in the lower Tilemsi valley (Manning et al. 2011; Manning and Fuller 2014; Neumann 2018). Earlier evidence in the form of ceramic temper from the desert of Northern Mali suggest a sequence of pre-domestication cultivation starting from 4000 to 5000 BC (Fuller et al. in press). This supports domestication in or around the Tilemsi valley and subsequent spread southwards into the savannas and through the Sahel. Finds from Ounjougou and Windé Koroji south of the Niger river, and Birimi in Ghana, attest to the rapid spread of pearl millet cultivation by ca. 1700 BC (D’Andrea et al. 2001; Ozainne et al. 2009; Manning et al. 2011). Nevertheless, the possibility of a second independent domestication of pearl millet in Mauretania associated with the Tichitt-Oualata Neolithic cultures remains plausible (MacDonald et al. 2009; Manning and Fuller 2014). Regardless of the number of origins, pearl millet was established since the Neolithic as the primary staple grain in West Africa, as it continued for many areas into modern times. Sadia fits into this long-term tradition.

Concerning sorghum, genetic analyses on modern sorghum and recent archaeobotanical evidence support domestication of this cereal in the Eastern Sahel region around central/eastern Sudan (Fuller and Hildebrand 2013; Fuller and Stevens 2018). Data from sorghum chaff and spikelet impressions in ceramics indicate that morphologically domesticated forms were close to 70% the population before 3000 cal BC around the upper Atbara river east of Khartoum (Winchell et al. 2017, 2018; Barron et al. 2020), with domestication fully established at Jebel Moya, ca. 2500 BC (Brass et al. 2019), although some communities continued to use a mixture of wild and domesticated sorghum, such as at Kasala (eastern Sudan) at ca. 1850 cal BC (Beldados et al. 2018). Already between 2000 and 1700 cal BC, sorghum appears to have been introduced to India (Fuller and Stevens 2018). The first sorghum evidence in West Africa is inferred to date by around 650 cal BC from Alibori sites in North Benin (Champion and Fuller 2018a, 2018b), and sorghum is reported from sites near Lake Chad in the later First Millennium BC, such as Mege and Kursakata (Klee et al. 2004; Bigga and Kahlheber 2011; Fuller and Stevens 2018). In the Inland Niger Delta in Mali, the earliest evidence of sorghum appears around 250 cal BC in Jenne-Jeno and starts to spread in the area around 500 cal AD. It is only around the tenth century AD that sorghum is found outside the Niger river delta and banks, such as at the sites of Sadia, Tongo Maaré Diabal and Oursi. By that period, sorghum starts to be more widespread in West Africa, but always in smaller proportion than pearl millet or rice.

#### Cowpea

A very important characteristic of cowpea, from an agronomic perspective, is that it is very fast growing and relatively easy to cultivate. It is a versatile crop that can be intercropped with pearl millet and sorghum (D’Andrea et al. 2007; Fuller 2003), and its seeds and leaves can be collected several times before the plant dies. As with other Fabaceae (legumes), it fixes atmospheric nitrogen and therefore helps to boost soil nitrates. Thus, its cultivation in rotations can to a degree substitute for a fallow or lengthen the cycle between fallows.

It was during the initial pearl millet dispersion period that cowpea (*Vigna unguiculata*) appears to have been domesticated as an addition to agriculture, evident from finds in Ghana dated around 1700–1500 cal BC and from the Nok Culture around 800 cal BC (D’Andrea et al. 2007; Franke 2016).

In Sadia, cowpea was probably intercropped with pearl millet and sorghum. However, cowpea remains are usually processed directly in the field and are therefore less common archaeological remains compared to pearl millet that was routinely processed in the village.

#### African rice

In the current state of research, the earliest evidence for *Oryza glaberrima* comes from the Inland Niger Delta at the site of Dia. AMS dates on rice grains coming from the earliest occupation layers of this settlement mound place them in the 800–400 cal BC bracket (Murray 2007). Further chronological resolution is difficult, as this falls in the Iron Age calibration plateau, and thus a date of fifth century BC is just as probable as eighth century BC. This rice is regarded as already domesticated, on the basis of rice grain measurements, as spikelet bases were not recovered (Murray 2007). Given these data, it is not clear whether an earlier domestication process in the Inland Niger Delta should be hypothesised (as suggested by Portères 1976), or whether rice had spread to this area from another domestication zone, such as the tributaries of the Niger River to the southwest, as suggested by modern genetic studies (Meyer et al. 2016). Interestingly, the first divergence within African rice is inferred to be between western Guinea (coast and hill) races and those of the Niger basin (Meyer et al. 2016). But currently, with the exception of the site of Juffure in Gambia, dated to 1650–1900 cal AD (Gijanto and Walshaw 2014) and from Sadia (this study), all the sites with domesticated African rice are coming from within the Niger River Basin itself, including the Inland Niger Delta and river banks, like the Dendi in North Benin (Bedaux et al. 1978; Champion and Fuller 2018a, 2018b; Murray 2007).

In this context, there are different ways to understand the presence of African rice in Sadia, where it first appears around 900 cal AD. While early crop-processing stages (threshing and winnowing) and the presence of definitive rice weeds can support local cultivation, weedy taxa present might represent a combination of wild gathered millets, weeds from dryland millet cultivation (e.g. of fonio) or weed contaminants present in semi-processed rice spikelets received though trade. At present, the archaeobotanical data are ambiguous as to whether or not rice cultivation was actually conducted immediately around Sadia.

More generally, rice is well attested during this period on several archaeological sites located within the nearby Inland Niger Delta (Dia, Jenné-Jeno, Thièl, Tato à Sanouna, Toguéré Galia, Toguere Doupwil). Therefore, its presence at Sadia could result from trade and exchange, along with other items that circulated between both parts of the Bandiagara escarpment at this time (Huysecom et al. 2015). More easily, rice could also come from the Sourou valley located around 50 km south-east of Sadia on the other side of the flat sandy Seno plain, at the current border with Burkina Faso. The Sourou Valley is a large but understudied region of rice cultivation (Zwarts et al. 2019; Bin 2009; Yaro 1998). The region forms a 560-km^2^ humid area where every type of rice is currently cultivated (irrigated, rainfed and mainly deep-water rice). Inhabitants from the Sadia region are still buying and consuming the Sourou rice (Huysecom and Mayor personal observations). Although we do not know the ancestry of rice cultivation in this wet region, it is thus possible that the rice found in Sadia originated from the Sourou valley, an amazingly rich rice granary region where archaeological mound sites similar to Sadia are present but were never studied (Huysecom and Mayor observation).

Finally, several variants of wild rice are found today at the foot of the cliff and on the Bandiagara Plateau, as well as in some depressions of the Seno Plain. Collection from the wild and local cultivation of rice is practiced today in the region (Selleger 2014; Huysecom and Mayor personal observations). The rice found in Sadia could therefore be the product of local cultivation, especially if climatic conditions were a bit wetter than today, as rainfed rice typically requires a minimum of 700–800 mm of rainfall (Portères 1959; Jacquot and Courtois 1987).

#### Weeds and/or gathered seeds

Weedy taxa are likely to enter the site through being harvested with crops, and then removed during various stages of crop-processing, alongside chaff (Hillman 1984; Jones 1987; Thompson 1996; Fuller and Weber 2005; Harvey and Fuller 2005). Small, light weed seeds tend to be removed early in processing with the initial threshing and winnowing, while larger and heavier seeds are removed in subsequent winnowing (or sieving) after dehusking, and those seeds closest in weight/size to crop grains must be removed by final hand-picking of processed grain.

With their intermediate size and weight, carpet weeds and button weeds can be expected to have contaminated rice spikelets after threshing and winnowing. They are both widely reported groups of weeds of dry tropical agriculture, such as in millet cultivation or in rainfed rice, both in Asia and in Africa (Fuller and Boivin 2009). *Commelina* spp. produce quite large and heavy seeds, so they are likely to be removed in final processing after dehusking, including final hand-picking of clean grain. They are also well-known weeds of rice cultivation of both upland rainfed and lowland irrigated systems (Moody 1989; Weisskopf et al. 2014; Portères 1976; Bezançon 1995). These three types of weed taxa would be expected to have contaminated rice spikelets (i.e. in the husk), a form in which crops were likely to be traded as the husks would protect the grains from various forms of spoilage during exchange. The high presence of these species is an argument for the local cultivation of rice in Sadia.

Large-seeded weedy grasses *Paspalum* and *Echinochloa* could be contaminants removed in the latter processing stages, but it is also possible that they were present as gathered foods. It is currently not possible to determine whether these grasses represent weeds of harvested rice, gathered (or even managed/cultivated) foods or a combination of both. These large-seeded weedy grasses cannot be used as indicators of rice cultivation. However, it is worth noting that *Oryza* and *Echinochloa* show different spatial distributions in Sadia (Fig. 8), which supports the idea of *Echinochloa* as an important gathered or cultivated resource in its own right.

#### Fonio

The most common “Fonio” cereal is *Digitaria exilis* (commonly named true fonio or white fonio) domesticated from *Digitaria longiflora* (Hilu 1997; Adoukonou-Sagbadja et al. 2007). Fonio is widely cultivated across western Africa, but generally is considered a minor crop that is presently in decline. The arduousness of fonio processing due to the small size of the seeds seems to be related to its progressive disappearance (Portères 1976; Cruz et al. 2011). Moreover, the seeds require laborious dehusking, unlike pearl millet which is free-threshing and thus easily processed to clean grain for cooking or flour making (Fuller and Weber 2005). On the other hand, brewing can be achieved with some or all grain remaining in their husks, and fonio beers are common.

Economic importance of fonio lies in its ability to grow very quickly in poor, especially shallow, soils, in contrast to the deeper-rooted tall crops of sorghum and pearl millet. Nevertheless, yields are quite low. A study in Nigeria reports fonio yields between 22 and 322 kg/ha (Aliero and Morakinyo 2001), whereas pearl millet yields can be expected to be between 300 and 900 kg/ha (Fuller et al. 2019). Consequently, the interest of fonio is its ability to give reliable production in a very short time, even in low yields, on poorly cleared or shallow soils, which are marginal for pearl millet or sorghum and too dry for rice. It may be grown, for example, after a couple seasons of pearl millet or sorghum, before a fallow cycle (see, e.g. Portères 1976; Burnham 1980; Cruz et al. 2011).

Currently, fonio cultivation is declining. Boureima Tessougué, one of our informants from Dimbal (a village currently located 6 km from Sadia), explained in 2018 (pers. comm.) that cultivators are sowing less and less fonio for different reasons: the difficulty of cultivation and processing, and the lack of *main d’oeuvre* due to seasonal migration of young people to urban centres. They maintain the practice more for tradition, to keep seeds for the following year, than for real nutritional needs. But the importance of fonio is also symbolic in the Dogon Country, having a high significance in cosmological tales and in various rituals linked to animist cults (Dieterlen 1955; Griaule 1948).

Currently, timing of fonio domestication is unclear. However, Portères suggested that it was probably domesticated in the Inland Niger Delta (Portères 1959; Portères 1976). Its geographically widespread but patchy distribution could be taken to imply that it was once continuously distributed, and might therefore be of great antiquity (Blench 2006). However, finds have been strikingly absent from most archaeobotanical studies of either Neolithic or Iron Age sites, leading to the alternative hypothesis that it was a late, secondary domestication associated with diversification of agriculture, sometimes in a context of urbanisation (Champion and Fuller 2019). So far, the only strong regional evidence of fonio is coming from the site of Kirikongo in Burkina Faso (100–1500 cal AD), where fonio grains compose 8% of the total assemblage. However, only preliminary results are available from this site (Gallagher et al. 2016). In the current state of knowledge, the adoption of fonio in Sadia seems to occur shortly after its domestication.

#### Echinochloa

The high frequency of *Echinochloa* in Sadia raises the possibility that this was a major food resource, although domesticated form is not known in West Africa. However, it is an important gathered resource for some current populations (Portères 1976; Harlan 1989) and namely in the Dogon Country (Selleger 2014). *Echinochloa colona* and *Brachiaria ramosa* are still largely collected on a large scale in the Niger Valley during rice harvesting (Boré 1983). Moreover, it has been found as a major grain in some archaeobotanical studies, as in Essouk in North Mali (Nixon et al. 2011), and in the Inland Niger Delta at Jenné-jeno (McIntosh 1995). Indeed, this is often a utilised grain throughout the broad West African and Sahelian regions (Harlan 1989; Mercuri et al. 2018b), as well as Asia (Yang et al. 2015).

At Sadia, due to the quantity (up to 9% of the assemblage for phase 3 and 7% for phase 2) and size of the seeds, it seems probable that they represent a gathered or even cultivated cereal. The three main *Echinochloa* species (*E. stagnina*, *E. pyramidalis* and *E. colona*) are common weeds. *E. stagnina* is the bourgou grass growing in deep water (up to 4 m of water) and is usually gathered at the same time as rice harvesting and as the *Paspalum* species that are difficult to separate from rice, like in the valley of Sourou. It is considered the most common weed for floating rice types. However, bourgou is more often gathered as fodder for animal stock than as a grain for human consumption. In the Inland Niger Delta, this plant is claimed to be the most useful of all wild plants, providing, in addition to food and drink, fodder, thatch, caulking for boats, vegetable salt after calcining which is used to make soap and indigo dye. Also, it is the most common plant (domesticated or wild) that populates the Niger River floodplain (Burkill 1985; Chevalier 1932; Dalziel 1937; Portères 1952). *E. colona*, jungle rice, is also a wild weedy grass commonly found in irrigation ditches or near waterways but never in deep water. More common in the Senegal valley than in the Niger valley, it is often misidentified as *E. stagnina*. It is used more for the grain than for the fodder. *E. colona* is so appreciated as a minor cereal that it is the object of a kind of proto-cultivation as in some areas like in Sierra Leone, where it is cultivated (Burkill 1985). Asian populations of this species are the presumed ancestors of domesticated Indian Sawa millet (De Wet et al. 1983). *E. pyramidalis* is a Sahelian grass widely gathered on river edges. It is a weed of sorghum and irrigated rice cultivation. All the species are usually prepared in a similar manner to rice and are sometimes used to produce beer (Burkill 1985; Portères 1959; Boré 1983; Dalziel 1937).

In conclusion, weeds present in archaeobotanical assemblages can often suggest evidence of local cultivation. This assumes that before being sold or stored, crops are separated from the weeds, and by consequence, the weed seeds remain at the site where they were processed. However, the remains of *Paspalum* and *Echinochloa* species cannot be used here as indicators of rice cultivation, being more likely valued resources, gathered or traded in their own right.

#### Hibiscus

*Hibiscus* flowers are often used as a tea like beverage called ‘Carcadet’ or ‘Bissap’, but the seeds and leaves are also used to prepare sauces (Burkill 1997). It is certainly native to the African savannahs, but little is known of its origins as a cultivar, although Fuller and Hildebrand (2013) suggested it might have originated in the Sudan region. Currently, in West Africa, pearl millet fields and garden edges are often demarcated by *Hibiscus sabdariffa* cultivation (Gissima or sorrel). Presence of *Hibiscus sabdariffa* remains at Sadia provides some early evidence for its use.

#### Tree and shrubs

Tree and shrubs at Sadia mainly include baobab (*Adansonia digitata*), marula (*Sclerocarya birrea*), jujube (*Ziziphus* sp.), shea butter (*Vittelaria paradoxa*), African grapes (*Lannea microcarpa*), tamarind (*Tamarindus indicus*) and marula (*Sclerocarya birrea*) remains. These species are useful for their fruits, but also provide shade and manure for gardens and fields, and are often found in agroforestry parklands, a savannah landscape in which a few species of mature trees occur, scattered through cultivated or recently fallowed fields. *Lannea microcarpa*, the African grape, would have grown as vines on these trees or even within the settlement on outside walls or fences. Such fruits would have been a seasonal supplement to diet, providing variety, micronutrients (like vitamins), handy snacks and vegetal fat.

### Sadia and the agricultural diversification of the Dogon Country

Interestingly, archaeobotanical results from Sadia indicate that during phase 0 (before the first to third century AD) and phase 1 (mid eighth to tenth c. AD), the only cultivated crop was pearl millet, which composed around half of the botanical assemblage. However, phase 0 is clearly not connected to the three other phases, as it is characterised by ceramics decorated with composite cord-wrapped roulettes, a décor typical of the late regional Neolithic period, which ends around 500 cal BC (Ozainne 2013; Huysecom et al. 2015). Thus, phase 0 may rather be related to the end of the initial wave of agricultural diffusion from the Tilemsi Valley, mainly characterised by the cultivation of pearl millet and the presence of chaff temper and cord-wrapped rouletted décors in the pottery production, along with domesticated livestock, mainly cattle remains.

Phase 1 may coincide with a later diffusion of agricultural population originating from the Tichitt area in Southern Mauritania, through the Inland Niger Delta, bringing mound site development and social complexity (Mayor et al. 2014; Champion and Fuller 2018a; Champion 2019). This wave is accompanied by the introduction of iron production technology (Robion-Brunner 2008) and more complex agricultural systems, i.e. mainly augmentation of production and crop diversification (Champion 2019; Champion and Fuller 2018a, 2018b). At Sadia, the ceramic assemblage also shows a marked transition from phase 1 to phase 2, with an increase of braided and folded strip roulettes décors and an abrupt decrease of composite roulettes (Huysecom et al. 2015).

Phase 2 witnesses the rise of agricultural complexity brought by the addition of four new domesticated crops: rice, fonio, sorghum and cowpea. The advent of rice, which could be related to trade as well as local production, possibly inspired by agricultural practices in the Inland Niger Delta, may echo the diversification of materials used for jewellery in the cultural sequence, suggesting the development of connections with regional urban trade centres along the Niger and Bani Rivers, such as Gao and Jenné-Jeno (Huysecom et al. 2015; Mayor et al. 2014; Truffa Giachet et al. 2020).

The emergence of these new staple crops (sorghum, fonio, African rice, cowpea) and pulses suggests that the expansion of the site is based on more diversified agricultural practices. Thus, phase 2 is interpreted as a period in which demand for increased grain production was met by some expansion of agriculture into marginal soils with low labour investment through the cultivation of “catch crops” of fonio, together with gathering of wild grain from open savannah grassland and fallows (i.e. *Echinochloa*). Environmental conditions are thought to remain stable, and generally wetter than later or modern periods, under conditions of the Medieval Climatic Anomaly (Nash et al. 2016; Mayor et al. 2005). Locally, climatic stability from phases 1 to 3 is supported by previously published wood charcoal analyses from Sadia (Huysecom et al. 2015). In other words, increasing population indicated by site size increase may have put pressure on established agricultural production and encouraged extensification and diversification. This expansion would have been focused on predictable availability of crops rather than increasing yields through intensification, a pattern that mirrors the model for initial African domestications posited by Marshall and Hildebrand (2002).

It is possible that other weedy grasses occurred as either weeds in fonio fields or part of wild grain gathering (i.e. narrow-grained *Digitaria* sp., *Paspalum* sp.), although their presence as weedy contaminants of rice cannot be ruled out. As already noted, *Digitaria* sp. (1%) and *Paspalum* sp. (<1%) could be present as rice weeds or as gathered volunteer grains in harvests of fonio or *Echinochloa* sp. gathering. In total, 3.5% of the phase 2 assemblage is characteristic of weeds that usual grow in association with rainfed or less wet rice. It is possible that they were cultivated locally around ponds, but if rainfall was insufficient to support rice cultivation locally, then rice would have been imported from wetter areas like the Inland Niger Delta or the valley of Sourou. These weeds would then be the result of removal of contaminants after trade, during local final stages of crop processing, in particular dehusking, final winnowing and hand-picking.

Phase 3, which immediately precedes the site’s abandonment, coincides with the rise of fonio cultivation, corresponding to a general west African pattern visible on most sites being abandoned around 1300–1400 cal AD (Champion 2019). This suggests a possible reduction in woodland cover and increasing cultivation of more marginal soils with fonio. Assuming that *Echinochloa* was gathered from wild grassland stands, its decrease could also result from an increase in fonio cultivation. Keeping in mind that the yields of fonio are perhaps only half or less than equivalent areas of pearl millet cultivation, caloric staple production through fonio would have required more land.

Wood charcoal analysis done by B. Eichhorn indicated a similar pattern of agricultural expansion through time. Indeed, the presence of *Prosopis africana*, *Terminalia* sp. and *Vitellaria paradoxa* (Shea butternut tree) wood charcoals corroborates that phase 0 is characterised by a savannah with open areas of pearl millet fields, in which the presence of fallow is inferred from the presence of *Terminalia* sp. that readily grow in old fallow. *Prosopis* sp. are characteristic of a savannah with limited agricultural influence. Wood charcoals from phase 1 seem to indicate an extension of the cultivation area with the rise of Shea butternut tree and decrease of *Prosopis africana*. During phases 2 and 3, the tree and shrub macro-remains dwindle, coinciding with a diminution of the useful tree species (*Vitex* sp., *Vitellaria* sp*.*) present in the wood charcoals. Moreover, the rise of old and young fallow species would indicate that the total land area under cultivation increased at this time, with more field area devoted to agriculture, some left uncultivated as long fallow, allowing for increased regeneration (*Detarium microcarpa*, *Pterocarpus erinaceus*, *Terminalia* etc.), and other areas with short fallow periods (*Guiera senegalesis*) (Huysecom et al. 2012, 2015).

**Fig. 9:**
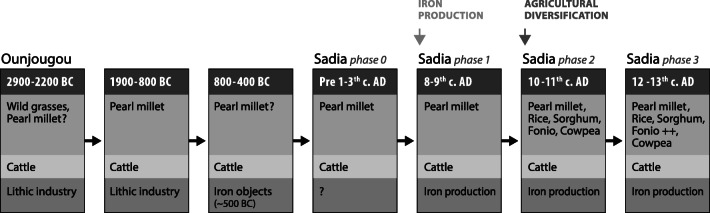
Summary of the Dogon Country late and terminal Holocene archaeological sequence with chronology and main characteristics of plant remains, domesticated animals and techniques

Not far from Sadia, on the Bandiagara plateau, the Ounjougou area provides a great number of Holocene archaeological sites excavated during the same project (Ozainne et al. 2014, 2017; Fig. 9). None of these sites were systematically sampled and analysed for macro-archaeobotanical purposes. Nevertheless, direct evidence of domesticated pearl millet cultivation dated to the 2nd millennium BC was found at the site of Varves–Ouest. Two pearl millet grains were directly dated to 1981–1494 cal BC (95.4% calibration) or 1692–1533 cal BC (Bayesian HPD) (Erl-9196; 3416±109 BP), and to 1628–976 cal BC (95.4% calibration) or 1285–1055 cal BC (Bayesian HPD) (Erl-9197; 3078±131 BP) (Ozainne et al. 2009). Moreover, bilobate phytoliths (characteristic of pearl millet phytoliths) from the Yamé River deposit dated to 2600–2300 BC may constitute indirect evidence of pearl millet cultivation, several hundred years before the appearance of its charred grains in the archaeological record (Garnier et al. 2013). Both direct and indirect indicators of pearl millet agriculture coincide with a radical change in ceramic tradition associated with the presence of numerous grinding stones and the appearance of settlement structures (Huysecom et al. 2004; Ozainne et al. 2009; Eichhorn and Neumann 2014). Such material culture and the presence of domesticated pearl millet coincide with contemporaneous similar finds at Windé Koroji around 2200–1900 cal BC (Macdonald et al. 2017). In the Ounjougou region, settlement mound sites occur from around 800 BC. From around 400 cal BC to 300 cal AD, a noticeable occupational decrease is noted on the Bandiagara plateau and the Seno Plain, the only evidence of human activities being recorded in a few caves of the Bandiagara cliff (Mayor et al. 2005, 2014; Huysecom et al. 2015; Fig. 9). The final Holocene (300–1500 cal AD) is marked by the growth of mound sites into larger mounds on the Seno plain, such as Sadia and the appearance of iron production sites on the plateau (Ozainne et al. 2017; Robion-Brunner 2008). The apogee of Sadia, around 900–1250 AD, ‘corresponds to the establishment of networks of villages, self-reliant in food resources and everyday consumption goods, alongside the first urban settlements along the Niger (e.g. Jenné-Jeno)’ (Ozainne et al. 2017: 223).

The long-term trend identified among the agro-pastoral community of Sadia indicates both the increase of agricultural production and the diversification of crops, with the consequence of bringing more land under cultivation, reducing the length of fallow, and removing or thinning woodlands. Wild resources were nevertheless important. During the apogee of the site, wild gathering of ‘kreb’ grasses, especially *Echinochloa*, became a prominent part of the subsistence economy. Local production was possibly also augmented with the cultivation of rice or its importation from wetter areas.

The agricultural diversification occurring at Sadia is probably the result of a combination of different factors. Firstly, the emergence of these new staple crops (sorghum, fonio, African rice, cowpea) coincided with the site expansion (from 1.5 ha in phase 2 to 3 ha in phase 3). We can suppose that this expansion was due to a rise of population, and in order to feed this growing population it was necessary to augment the food production and to explore new land (as the most suitable would have already been used) and new crops that are adapted to these new fields.

In that case, the expansion of the site is due to the emergence of new specialised cultivators. For the Inland Niger Delta, R. McIntosh developed the ‘pulse model’ theory and hypothesised that urbanisation and the growth of site size was supported by the emergence of specialist groups including agricultural specialists that could sustain a larger population. In the case of Sadia, these agricultural specialists developed a more diversified agriculture and developed new skills in the utilisation of local plants (e.g. *Echinochloa*, fonio) and local ecological niches.

Secondly, the utilisation of new soil (more marginal soil) with the adoption of new cultivars (cowpea, fonio, Echinochloa) could also result from growing insecurity (ecological and social) that pushed people to explore agricultural risk buffering. Indeed, Sadia’s growth and abandonment coincide with a more general trend observed in Sahelian West Africa where most of the sites collapsed between 1200 and 1500 AD (Mayor et al. 2005; Mayor 2011). From abrupt climate changes, to war (i.e. the Songhay invasion) and diseases (i.e. the plague the ravaged Europe in the 1300s), several reasons are proposed for this sub-continental settlement rupture (Breunig 2013; Macdonald 2013; McIntosh 1993, 2000). However, no strong evidence can confirm or refute any of these potential causes. Nevertheless, all these factors could have contributed to insecurity/instability that pushed towards both more specialised and more diversified agriculture. Although there is a lack of high precision climatic proxies near Sadia, the wider western African region appears to have experienced wetter conditions during the Medieval Climatic Anomaly (900–1250 AD) after which aridity set in (Nash et al. 2016). This could plausibly contribute to Sadia’s decline. In contrast, whereas a role for the plague pandemic in west Africa for abandonments of many sites from ca. 1350–1500 AD has been recently explored (Chouin 2018; Gallagher and Dueppen 2018), this is later than the abandonment of Sadia (ca. 1300 AD). Nevertheless, some inherent declines in soil fertility as result of agricultural expansion and accumulating soil exhaustion over the course of phases 2 and 3 could also have contributed to the site’s decline.

### Sadia in its regional context

So far, archaeobotanical analyses have provided data on agricultural practices contemporary with the Sadia occupations in four neighbouring regions.

To the west of Sadia, the sites from the Inland Niger Delta (Dia, Jenne-Jeno, Toguere Doupwil, Toguere Galia) indicated agricultural practices mainly based on the use of African rice (which usually dominates), coupled to pearl millet, sorghum and fonio cultivation. Niger Delta agriculture was based mainly on wetland plant cultivation, complemented by those of dry land cultivation. Indeed, the archaeobotanical assemblages are largely dominated by samples that contain domesticated rice (*Oryza glaberrima*), *Echinochloa colona* and wild *Brachiaria* cf. *ramosa*. *Brachiaria* cf*. ramosa* is an annual wild grass that grows in very similar ecological conditions to floodplain rice (but not deepwater rice). The secondary agricultural practices are related to the cultivation of dry crops such as pearl millet (*Pennisetum glaucum*) and fonio (*Digitaria exilis*). Like in the Sadia region, pearl millet is present from the first millennium BC, and fonio is often more common on settlements during their final phases and just before the site’s abandonment. The presence of sorghum, a décrue cereal, is also notable, but only present in very small quantities.

To the north-east, the site of Tongo Maaré Diabal located in the north of the Dogon Country shows a very different archaeobotanical assemblage. Indeed, the foods consumed at this site included mainly pearl millet (up to 85%) and wild millet species (*Digitaria* sp.). The sporadic consumption of fruits of local trees (*Sclerocarya birrea* and *Vitex* sp.) is also indicated. These are recovered in low quantity (3% frequency in average). The proportion of various plant remains at Tongo Maaré Diabal appears little changed throughout the occupation. The introduction of sorghum is noticed from phase 3 (750–900 AD) until the abandonment phase (1000–1150 AD), but always in very small quantity.

The absence of agricultural diversification at Tongo Maaré Diabal is also visible through isotope analyses. Recent carbon and nitrogen isotope analysis on human teeth from people buried in the Bandiagara escarpment indicate diets with a major input of C4 foods (e.g. pearl millet) in the region of Tongo Maaré Diabal, and more diversified diets mixing C3 (e.g. rice and cowpea) and C4 foods in the southern part of the Bandiagara escarpment, close to Sadia. There, a temporal shift from a C4 food system to a mix system (C3 and C4) is also observed (Dlamini et al. 2019) and is convergent with our archaeobotanical results (Fig. 6, Table 1).

In the same way, nitrogen isotopes on pearl millet grains from Tongo Maaré Diabal show a lack of increase in pearl millet δ15N values during the 650-year occupation of the site, implying that manure/household waste was added only sporadically to pearl millet (Styring et al. 2019). In other words, manuring was not a normal practice for continued annual processing, suggesting instead fallowing cycles. This continuity in soil maintenance practice reflects the general lack of change in the botanical and charcoal assemblages.

Thirdly, to the east, in the Oursi region in Burkina Faso, archaeobotanical evidence is available since the Late Stone Age. Late Stone Age (Neolithic) Oursi inhabitants were probably seasonal agro-pastoralists, who were coming into this zone during the dry season and hunting wild animals and cultivating pearl millet. From 400 cal BC, permanent settlement mounds started to appear and grow. Economic subsistence was based on pearl millet cultivation. Cowpea was added to this agricultural system around 1 cal BC/AD, and sorghum around 500 cal AD, but in very low quantity. Throughout the sequence, fruit and wood charcoal remains of *Adansonia digitata*, *Sclerocarya birrea*, *Balanites aegyptiaca* and *Vitellaria paradoxa* suggest the presence of an agro-parkland system. Charred wood results illustrate that cultivation and settlement activities intensified considerably during the course of the occupation. For example, the system of fields and fallows, typical for this cultural landscape, replaced the natural *Acacia* savannah on the dune, as evidenced by the decrease of *Acacia* and the increase of Combretaceae charcoals (Höhn and Neumann 2012).

Finally, to the south, the site of Kirikongo also provides archaeobotanical data. This settlement site is composed of mounds representing household compounds. This site complex was founded around 100 cal AD and abandoned like many other sites around 1400–1500 cal AD, possibly including the impact of plague (Gallagher and Dueppen 2018). Inhabitants were performing a large array of economic tasks, from herding cattle, producing and forging iron, potting and cultivating. The most ubiquitous and numerically frequent plant taxon at the site is Shea Butter nut (*Vitellaria paradoxa*). The large quantity of carbonised shea fragments is attributed to the use of parching ovens, one of the steps in Shea butter production (Gallagher et al. 2016). Agricultural activities were based on pearl millet and fonio cultivation. Inhabitants were also collecting wild grasses and arboricultural products (baobab and shea butter). Archaeobotanical samples from the different settlement mounds is not fully published but initial results attest to the presence of sorghum from phase red II (1100–1260 cal AD) (Gallagher et al. 2016).

In addition, we should point out that large areas, such as the Sourou Valley, have never been the subject of archaeological research, even though important tell sites have been identified.

In brief, West African Sahelian sites contemporaneous from Sadia’s occupation usually show a high dominance of pearl millet. But from early Medieval times, the sites along the Niger River valley show a specific pattern, with a much more diversified agriculture than the sites located in the dry margins, such as the Oursi region sites or Tongo Maare Diabal in the northern part of the Dogon Country. Despite its rural remote location in the Seno plain, Sadia’s sequence evidence an agricultural diversification paralleling the Inland Niger Delta’s economy.

## Conclusion

The archaeobotanical sequence at Sadia in Central Mali reveals the expected long-term dominance of pearl millet as a local staple crop in a landscape that resembles a managed parkland with many useful trees producing edible fruits. But diversification of agriculture is evidenced around the tenth c. AD with the adoption of sorghum, fonio and rice, the latter in the form of grain importation from the closest wetlands in the Inland Niger Delta or in the Sourou valley, or a local production inspired by the practices of these regions. In addition, evidence suggests some use of wild gathered savanna grains, a traditional component of subsistence found widely through the Saharan and Sahelian regions. In this context, grain crop diversification over time may correspond with an increasing use of land for field crops and decreasing persistence of useful savannah trees.

If we consider wider time and space scales, agriculture in the Dogon country seems to have developed in two separate steps. The first step, dating from 1900 to 1600 BC until 800–400 BC was based on pearl millet cultivation, probably coming from the Tilemsi Valley and recognised also in Winde Korodji in the northern part of the Dogon country. As in many West African areas, this period was followed by a cultural hiatus between around 400 BC and 300 AD, with no evidence of cultivation in the area, despite sporadic occupation recognised in the Bandiagara caves. After this hiatus, the second step coincides with a renewed pearl millet cultivation, the beginning of iron production and the development of village networks connected to important proto-urban trade centres. This might be related to influences from Southeast Mauretania associated with the Late Tichitt tradition and the setting of the Ghana empire. As in the neighbouring Inland Niger Delta, this led to a diversified agricultural system that included several domesticated crops (rice, sorghum, fonio and cowpea).

Previously, fonio was often considered a very ancient crop, with widespread and patchy modern distributions reflecting relict populations. Alternatively, we support the hypothesis that the fonios are later secondary domesticates, taken into cultivation to complement pearl millet, rice and sorghum, as risk-buffering crops for poor and shallow soils, plausibly in more than one time and place. The fonio millets may have been taken up secondarily to suit the more marginal agricultural conditions across the West African dry savannas and to help expand grain crop production as population centres grew. In this model, the evidence for fonio in the Seno plain at Sadia may be seen as a local development of cultivation at the interstices of established pearl millet agriculture and early rice expansion along the Niger river.
